# Comparative safety profile of levofloxacin versus moxifloxacin in first-line tuberculosis therapy: a pharmacovigilance study of the FAERS database

**DOI:** 10.3389/fphar.2025.1713170

**Published:** 2025-11-21

**Authors:** Yanlang He, Lifen Liang, Sheng Wei

**Affiliations:** 1 Department of Infectious Disease, Shaoyang Central Hospital, Shaoyang, China; 2 Department of General Medicine, The Second Affiliated Hospital of Wannan Medical College, Wuhu, China

**Keywords:** safety, fluoroquinolone, tuberculosis, adverse event, FAERS

## Abstract

**Objective:**

This study aimed to compare the safety profiles of two fluoroquinolone-containing regimens, HREL (isoniazid, rifampicin, ethambutol, levofloxacin) and HREM (isoniazid, rifampicin, ethambutol, moxifloxacin), in the treatment of drug-susceptible tuberculosis (DS-TB), focusing on adverse events (AEs) across age and gender subgroups.

**Methods:**

Data were extracted from the FDA Adverse Event Reporting System (FAERS) database (2004–2024). Disproportionality analyses were conducted to assess AE signals for HREL and HREM. Pharmacovigilance metrics, including reporting odds ratios (RORs), proportional reporting ratios (PRRs), Bayesian confidence propagation neural networks (BCPNN), and empirical Bayesian geometric means (EBGMs), were calculated. Subgroup analyses were stratified by age (≥60 vs. <60 years) and gender.

**Results:**

The analysis included 451 HREL-related and 338 HREM-related AEs. HREL was associated with a significantly higher risk of immune reconstitution inflammatory syndrome (IRIS-TB) and drug-induced liver injury, particularly in females and patients under 60 years of age. In contrast, HREM demonstrated a higher risk of drug reaction with eosinophilia and systemic symptoms (DRESS), peripheral neuropathy, and severe hepatobiliary events such as acute liver failure. Subgroup analyses revealed that these risk profiles were influenced by age and gender. Specifically, elderly patients (≥60 years) receiving HREM had a lower risk of IRIS-TB but a higher propensity for severe liver injury. Male patients treated with HREM showed an increased risk of neurological events, including thalamic infarction.

**Conclusion:**

HREM may be preferred for elderly patients (≥60 years) due to a lower risk of immune-related events like IRIS-TB, but requires careful liver monitoring. HREL could be an option for younger patients (<60 years), though vigilance for hepatotoxicity and IRIS-TB is needed, especially in females. For males on HREM, increased attention to neurological AEs is recommended.

## 1 Introduction

Tuberculosis (TB) is a global public health issue caused by *Mycobacterium tuberculosis*, posing a significant threat to human health. According to the World Health Organization’s latest Global Tuberculosis Report 2024, there were an estimated 10.8 million incident cases of TB globally in 2023, resulting in 1.25 million deaths, making it the second leading infectious disease after COVID-19 (World Health Organization, 2024). Tuberculosis can be categorized into drug-susceptible TB (DS-TB) and drug-resistant TB, with DS-TB being the predominant form, accounting for the vast majority of all TB cases. As recommended by the World Health Organization, the standard regimen for the intensive phase treatment of all forms of DS-TB is HRZE, which consists of isoniazid (H), rifampicin (R), ethambutol (E), and pyrazinamide (Z). This regimen has achieved successful treatment outcomes in approximately 85% of patients, thereby saving millions of lives (World Health Organization, 2022). Although the latest Centers for Disease Control guidelines now recommend the shorter 4-month rifapentine-moxifloxacin-based regimen (4-HPMZ) (Carr et al., 2022), its adoption remains limited. Few tuberculosis programs have implemented 4-HPMZ, and only one retrospective cohort study with a small sample size and limited demographic and clinical information has reported outcomes (Louie et al., 2024). Furthermore, challenges such as drug shortages, cost concerns, and potentially higher rates of adverse events (AEs) have been noted with this regimen (Wilson et al., 2025). Consequently, the 6-month regimen remains the primary treatment for DS-TB.

However, the tolerability of pyrazinamide in this regimen has long been a challenge in clinical practice. Studies have shown that the incidence of pyrazinamide-related serious AEs is significantly higher in elderly patients than in younger patients, with hepatotoxicity, gastrointestinal discomfort, skin reactions, and arthralgia being the most common adverse effects (Kwon et al., 2020). These adverse reactions severely affect treatment adherence and continuity in the elderly population. Similarly, although to a lesser extent, these adverse effects are also present in younger patients and should not be overlooked. Some guidelines suggest that pyrazinamide should be avoided in elderly tuberculosis patients over 75 years of age with moderate disease and low risk of drug resistance, as the potential risks may outweigh the benefits in this population (Nahid et al., 2016). However, this recommendation is not applicable to patients with severe tuberculosis, such as those with cavitary pulmonary tuberculosis, as it may prolong unnecessary treatment duration or increase the risk of treatment failure. The American Thoracic Society and the Centers for Disease Control and Prevention recommend that for patients with severe lesions who cannot tolerate pyrazinamide, a fluoroquinolone can be used as an alternative during the intensive phase of treatment (Nahid et al., 2016), which is consistent with previous evidence. Numerous clinical studies have confirmed that fluoroquinolones possess early bactericidal activity, capable of rapidly reducing the tuberculosis bacillary load, improving treatment outcomes (Johnson et al., 2006), and potentially shortening the duration of tuberculosis treatment (Pettit et al., 2023; Dorman et al., 2021).

Despite the widespread application of this concept in clinical practice, there is still ongoing debate regarding which fluoroquinolone should be used as the standard regimen. Moxifloxacin (M) and levofloxacin (L) are the most commonly used fluoroquinolones in tuberculosis treatment. Therefore, our study aims to compare the safety of these two fluoroquinolones in the treatment of drug-susceptible tuberculosis patients using the FAERS (FDA Adverse Event Reporting System) database, thereby providing reference for the optimal use of HREM (H, R, E, M) or HREL (H, R, E, L) regimens in anti-tuberculosis therapy.

## 2 Methods

### 2.1 Data source

The FAERS is an online database maintained by the FDA. Since its launch in 2004, it has collected reports of drug adverse reactions, medication errors, and product quality complaints submitted in the form of individual case safety reports by healthcare professionals, manufacturers, and patients from over 150 countries and regions, including the United States (Zhou et al., 2022). Due to the variability in data structure between quarters and the lack of some variables, these cases are typically standardized to fit a uniform structure. FAERS utilizes the most current edition of the Medical Dictionary for Regulatory Activities (MedDRA®) to accurately encode each adverse drug reaction (ADR) and employs the WHO’s Anatomical Therapeutic Chemical (ATC) codes to standardize drug nomenclature. The FAERS database includes eight types of files: demographic and administrative information (DEMO), drug information (DRUG), adverse events (REAC), patient outcomes (OUTC), report sources (RPSR), start and end dates of the reported drug (THER), indications for use (INDI), and invalid reports (deleted). Each file contains the variables “primaryid” and “caseid,” which allow us to obtain specific information about patients and AEs through these variables. All files can be found on the FDA website at https://fis.fda.gov/extensions/FPDQDE-FAERS/FPD-QDE-FAERS.html. H, R, and E are fixed components of the intensive phase treatment for drug-susceptible tuberculosis. By bundling fluoroquinolones with these agents for analysis, we can ensure that the study is conducted among patients with drug-susceptible tuberculosis. We selected the two most widely used fluoroquinolones in current clinical practice (levofloxacin (L) and moxifloxacin (M)) and collected AE reports related to the use of HREM and HREL regimens from the FAERS database. Since these data are publicly available and open to anyone, the requirement for informed consent and approval from an institutional review board is waived.

### 2.2 Data processing

The study downloaded ASCII report files from the FAERS database covering the period from July 1, 2004, to September 30, 2024. The data were then imported and processed using RStudio (version 4.2.2). Following the FDA’s recommendations, a two-step duplicate removal process was used to ensure the uniqueness of the reports: 1) selecting the higher “primaryid” when “caseid” and “fda_dt” are the same, and 2) selecting the latest “fda_dt” when “caseid” is the same. Here, “primaryid” is the unique identifier for an adverse drug reaction (ADR) report, “caseid” is the unique identifier for a case, and “fda_dt” is the date the FDA received the case. Data were subsequently extracted from the DELETED files in the FAERS database to remove invalid reports and obtain the final set of reports. Reports with incorrect dates (drug use time later than the event occurrence time) and those with missing date data were also excluded. The hierarchical structure of the Medical Dictionary for Regulatory Activities (MedDRA, version 25.0) is divided into five levels: System Organ Class (SOC), High-Level Group Term (HLGT), High-Level Term (HLT), Preferred Term (PT), and Lowest Level Term (LLT). In FAERS pharmacovigilance analyses, AEs are encoded using the PT of MedDRA. A specific PT can be assigned to a SOC. All relevant PTs generated by the use of HREM and HREL regimens were analyzed, with an inclusion criterion of ≥3 PT event counts.

To ensure the cohort specifically represented patients exposed to the target multidrug regimens, case selection was performed with the following criteria. A case was included in the HREL cohort only if it contained reports for all four constituent drugs: isoniazid, rifampicin, ethambutol, and levofloxacin. Similarly, a case was included in the HREM cohort only if it contained reports for isoniazid, rifampicin, ethambutol, and moxifloxacin. Cases reporting any additional anti-tuberculosis drugs beyond these core combinations were excluded to maintain regimen specificity and minimize confounding. The role code of AEs is designated by the reporter, including Primary Suspect (PS), Secondary Suspect (SS), Concomitant (C), and Interaction (I). Our analysis was restricted to reports with “role_cod” designated as “PS” in the DRUG file (Zhou et al., 2022; Mascolo et al., 2021; Moore et al., 2022). To ensure that the analyzed AEs were those most directly attributed by the reporter to the fluoroquinolone, the analysis was restricted to reports where the fluoroquinolone component (i.e., levofloxacin for HREL or moxifloxacin for HREM) was designated as the PS drug. Epidemiological baseline information was extracted from these reports, including patient age, reporting country, reporter type, report date, route of administration, outcome, and the timing of drug-related AE occurrence.

### 2.3 Disproportionality analysis

In pharmacovigilance research, disproportionality analysis is a widely used data mining method globally. It assesses the association between drugs and AEs by comparing the observed frequency ratios in exposed versus non-exposed populations using contingency tables (Table 1). We employed this method to identify AEs associated with the HREM and HREL regimens. In this study, we calculated the Reporting Odds Ratio (ROR), Proportional Reporting Ratio (PRR), Bayesian Confidence Propagation Neural Network (BCPNN), and Empirical Bayes Geometric Mean (EBGM). The PRR provides higher specificity (Evans et al., 2001), while the ROR corrects for biases arising from low reporting numbers (Rothman et al., 2004). The EBGM is used for detecting signals of rare events, and the BCPNN performs well in integrating multiple data sources and conducting cross-validation (Bate et al., 1998). To enhance signal reliability and avoid false-positive signals, a signal was considered relevant only when it met the criteria of all four algorithms simultaneously (Shu et al., 2022). Typically, the algorithm scores are higher when the target drug is more likely to induce the target AE compared to all other drugs. Additionally, reference to previous similar studies (Liu et al., 2025; Li et al., 2025; Yang et al., 2024), we adjusted the thresholds and variances to detect more rare AEs. Specifically, by lowering the threshold for all algorithms, we proactively incorporated potential signals that would have been excluded due to high variance back into our scope of scrutiny. This enables our analysis to better capture rare AEs that have low reporting frequencies but potentially very high association strengths. The specific formulas and thresholds for all algorithms are detailed in Table 2. Data extraction was performed using MySQL 8.0, and statistical analyses were conducted using RStudio (version 4.2.2).

**TABLE 1:** Four-grid table for signal detection.

Research object	Drug-related ADEs	Non-drug-related ADEs	Total
Drug	a	b	a+b
Non-drug	c	d	c+d
Total	a+c	b+d	N=a+b+c+d

ADE, adverse drug events. a is the number of cases where a specific adverse event occurred after using HREL/HREM, b is the number of cases where HREL/HREM, were used but the specific adverse event did not occur, c is the number of cases where the specific adverse event occurred without the use of HREL/HREM, d is the number of cases where neither HREL/HREM, were used nor the specific adverse event occurred.

**TABLE 2:** Four main algorithms are used to evaluate the correlation between HREL/HREM and AEs. This includes ROR, PRR, BCPNN, and EBGM methods, formulas, and thresholds.

Method	Formula	Threshold
ROR	$ROR=\left(a\;/\;c\right)/\left(b\;/\;d\right)$	a ≥3
	$95\%CI=e^{\ln\left(ROR\right)\pm 1.96\sqrt{\left(\frac{1}{a}+\frac{1}{b}+\frac{1}{c}+\frac{1}{d}\right)}\;}$	ROR ≥2 95%CI (lower limit) > 1
	
PRR	$\text{PRR}=a\left(c+d\right)/c/\left(a+b\right)$	a ≥3
	$95\%CI=e^{\ln\;\left(\text{PRR}\right)\pm 1.96\sqrt{\;\frac{1}{a}-\frac{1}{a+b}+\frac{1}{c}-\frac{1}{c+d}\;}}$	PRR ≥2 95% CI (lower limit) > 1
	
BCPNN	$IC=\log_{2}\frac{a\left(a+b+c+d\right)}{\left(a+b\right)\left(a+c\right)}$	IC025 > 0
	$\gamma =\gamma ij\frac{\left(N+\alpha \right)\left(N+\beta \right)}{\left(a+b+\alpha i\right)\left(a+c+\beta j\right)}$ $E\left(IC\right)=\log_{2}\frac{\left(a+\gamma ij\right)\left(N+\alpha \right)\left(N+\beta \right)}{\left(N+\gamma \right)\left(a+b+\alpha i\right)\left(a+c+\beta j\right)}$	
	$V\left(IC\right)=\frac{1}{{\left(\ln2\right)}^{2}}\left[\frac{\left(a+b+c+d\right)-a}{a\left(1+a+b+c+d\right)}+\frac{\left(a+b+c+d\right)-\left(a+b\right)}{\left(a+b\right)\left(1+a+b+c+d\right)}+\frac{\left(a+b+c+d\right)-\left(a+c\right)}{\left(a+c\right)\left(1+a+b+c+d\right)}\right]$	
	$IC025=E\left(IC\right)-2\surd \left(V\left(IC\right)\right)$	

EBGM	$EBGM=\left(a\left(a+b+c+d\right)\right)/\left(\left(a+c\right)\left(a+b\right)\right)$	EBGM05 > 2
	$95\%CI=e^{\ln\left(EBGM\right)\pm 1.96\sqrt{\left(\frac{1}{a}+\frac{1}{b}+\frac{1}{c}+\frac{1}{d}\right)}\;}$	


N, the number of reports; a is the number of cases where a specific adverse event occurred after using HREL/HREM, b is the number of cases where HREL/HREM, were used but the specific adverse event did not occur, c is the number of cases where the specific adverse event occurred without the use of HREL/HREM, d is the number of cases where neither HREL/HREM, were used nor the specific adverse event occurred; ROR, reporting odds ratio; γ, γ_ij_ represent the parameters of the Dirichlet distribution; α, α_i_, β, β_j_ represent the parameters of the Beta distribution; SD, standard deviation; ROR: reporting odds ratio, PRR: proportional reporting ratio, BCPNN: bayesian confidence propagation neural network, EBGM: empirical bayesian geometric mean; χ2, chi-squared; IC, information component; IC025, the lower limit of 95% CI, for the IC; E (IC), the IC, expectations; V(IC), the variance of IC; EEBGM05, the lower limit of the 95% CI, for EBGM; N: number of reports.

### 2.4 Time-to-onset analysis

First, we excluded reports with input errors (e.g., EVENT_DT before START_DT), inaccurate data, or missing information. Next, the time to event onset was calculated as the difference between EVENT_DT (date of AE occurrence) and START_DT (date of drug initiation). We analyzed the time to event onset associated with AEs from the drug combination regimens and described this time interval using the median and interquartile range.

### 2.5 Data visualization

The charts were generated using the ggplot2 package and GraphPad Prism version 8.0.1. We used a world heatmap to visualize the data from countries/regions that submitted reports and employed line charts to illustrate the number of cases from 2004 to 2024. Additionally, we created various scatter plots based on the study results to compare the differences in AE occurrences between the two drug regimens.

## 3 Results

### 3.1 Basic information of AEs

**FIGURE 1:**
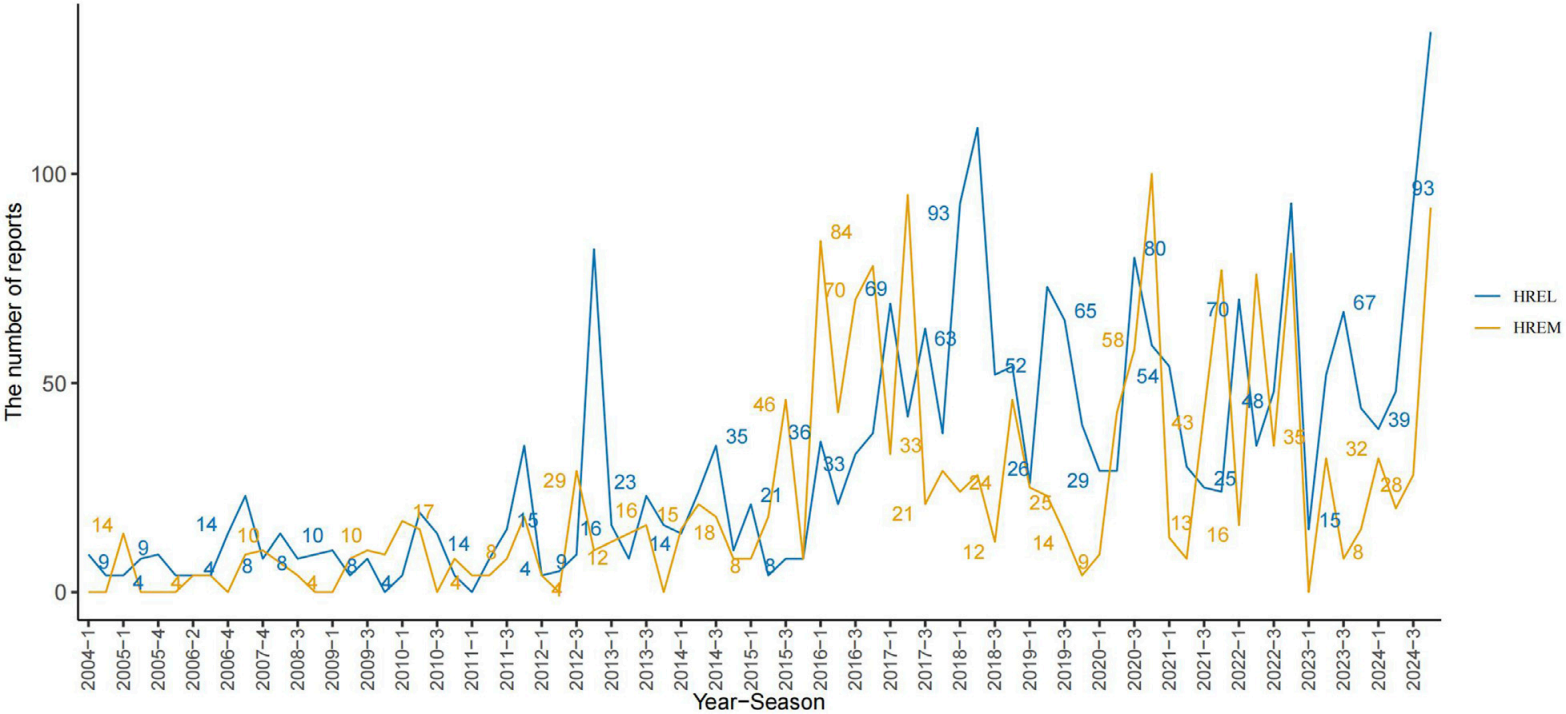
Trends in HREL and HREM Adverse Event Reports (2004‐2024).

**FIGURE 2:**
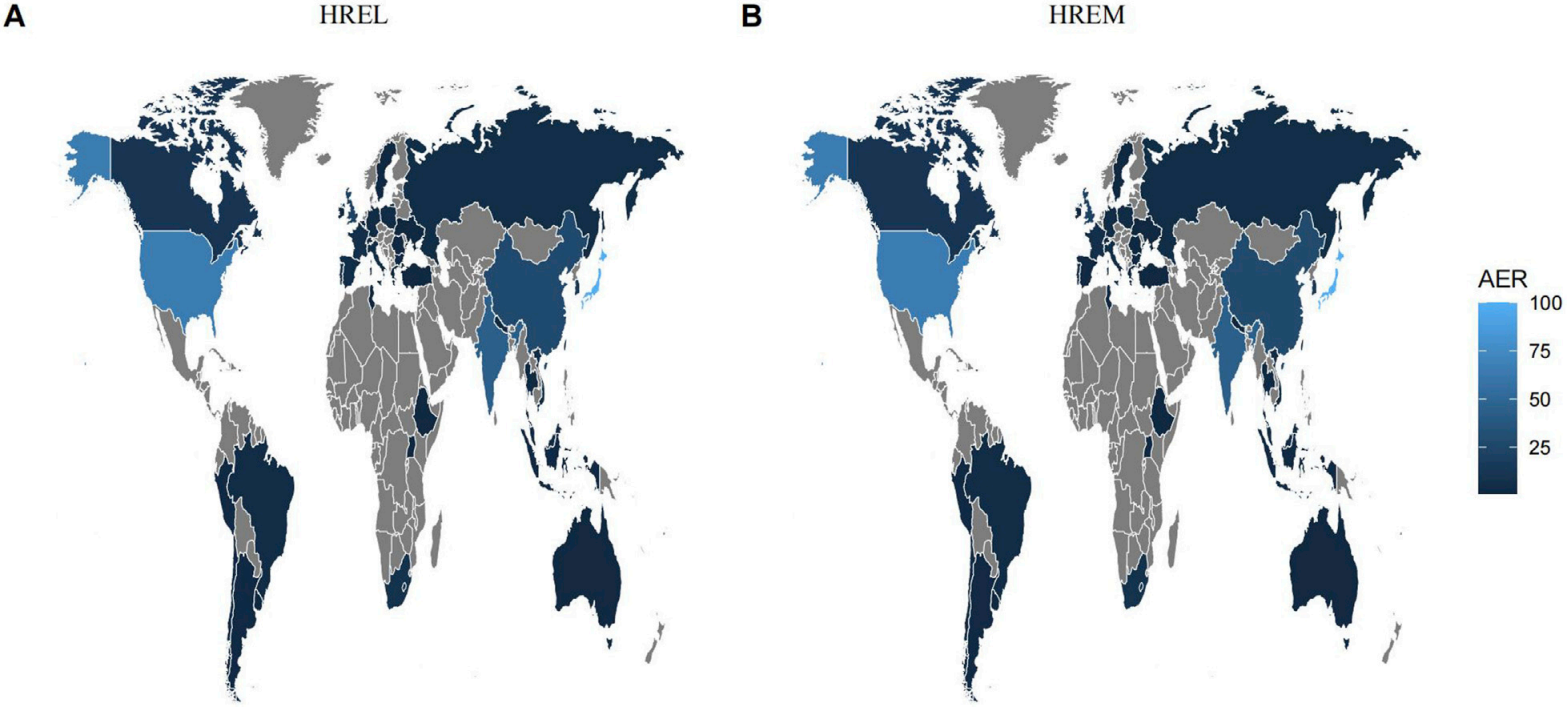
Global Distribution of Adverse Event Reporting Rates for HREL and HREM.

Detailed epidemiological features are shown in Table 3. In this study, we analyzed the treatment outcomes and demographic characteristics of 451 and 338 patients treated with HREL and HREM, respectively. The AEs for both regimens showed a gradual increasing trend, reaching a peak in 2024 (Figure 1). The median age of patients in the HREL group was 50 years, compared to 42 years in the HREM group, with interquartile ranges of 31–65 years and 33–57 years, respectively. The majority of reports were submitted by other healthcare professionals (169 in the HREL group and 100 in the HREM group), followed by pharmacists and physicians. Reports contributed by consumers were the least, with 16 in the HREL group and 19 in the HREM group. Regarding outcomes, other serious outcomes were most frequently reported (329 in the HREL group and 232 in the HREM group), followed by hospitalization and death. Geographically, Figure 2 shows the global distribution of AE reports. Reports with a specified country of origin were mainly from Japan and the United States, with 100 and 65 cases in the HREL group, respectively. All 338 cases in the HREM group had no specified country of origin, which limits our understanding of the geographic distribution of AEs. The route of administration was mostly unknown for most AEs. In terms of gender distribution, the number of female reports was similar between the HREM group (145) and the HREL group (160), while the number of male reports was higher in the HREL group (214) than in the HREM group (175).

**TABLE 3:** Epidemiological characteristics of AE reports.

Characteristics	HREL	HREM
Age_yr	50.00 (31.00,65.00)	42.00 (33.00,57.00)
Age_yrQ		
<60	221 (49.00)	230 (68.05)
≥60	132 (29.27)	71 (21.01)
unknow	98 (21.73)	37 (10.95)
Reporter		
Other health-professional	169 (37.47)	100 (29.59)
Pharmacist	131 (29.05)	116 (34.32)
Physician	121 (26.83)	99 (29.29)
Consumer	16 (3.55)	19 (5.62)
unknown	14 (3.10)	4 (1.18)
Outcomes		
other Serious	329 (51.81)	232 (53.46)
hospitalization	198 (31.18)	129 (29.72)
death	64 (10.08)	40 (9.22)
life Threatening	34 (5.35)	26 (5.99)
disability	10 (1.57)	6 (1.38)
congenital Anomaly	NA	1 (0.23)
Reported countries		
Other (Country information was missing, unreported, or not specifically classified)	234 (58.65)	338 (100.00)
Japan	100 (25.06)	NA
United States	65 (16.29)	NA
Route		
Other (Routes of administration other than oral or intravenous, or routes that were unknown)	422 (71.28)	290 (71.43)
oral	137 (23.14)	98 (24.14)
intravenous	33 (5.57)	18 (4.43)
Sex		
female	160 (35.48)	145 (42.90)
male	214 (47.45)	175 (51.78)
unknown	77 (17.07)	18 (5.33)
TTO	18.00 (4.75.45.50)	22.00 (10.00.54.00)
TTOQ		
0–31	35 (20.96)	61 (33.52)
31–61	19 (11.38)	17 (9.34)
61–91	5 (2.99)	1 (0.55)
91–121	0 (0.00)	9 (4.95)
121–150	0 (0.00)	2 (1.10)
151–181	0 (0.00)	1 (0.55)
181–361	4 (2.40)	6 (3.30)
≥361	3 (1.80)	3 (1.65)
unknow	101 (60.48)	82 (45.05)
Wt	52.00 (45.00.60.00)	52.10 (42.25.70.75)
Year		
2004	3 (0.67)	NA
2005	5 (1.11)	3 (0.89)
2006	5 (1.11)	2 (0.59)
2007	6 (1.33)	3 (0.89)
2008	7 (1.55)	2 (0.59)
2009	5 (1.11)	5 (1.48)
2010	8 (1.77)	7 (2.07)
2011	9 (2.00)	7 (2.07)
2012	10 (2.22)	9 (2.66)
2013	12 (2.66)	9 (2.66)
2014	15 (3.33)	13 (3.85)
2015	9 (2.00)	13 (3.85)
2016	29 (6.43)	40 (11.83)
2017	39 (8.65)	30 (8.88)
2018	57 (12.64)	24 (7.10)
2019	35 (7.76)	14 (4.14)
2020	39 (8.65)	40 (11.83)
2021	31 (6.87)	26 (7.69)
2022	36 (7.98)	41 (12.13)
2023	34 (7.54)	10 (2.96)
2024	57 (12.64)	40 (11.83)

### 3.2 Analysis by SOC level

In our pharmacovigilance analysis, using a ROR exceeding 2 as the criterion, we identified SOCs significantly associated with HREL and HREM. For hepatic and biliary disorders, HREL showed a significant ROR of 9.74 (95% CI 8.29, 11.45), while HREM also exhibited a significant ROR of 9.37 (95% CI 7.65, 11.49), highlighting a strong association for both regimens at this SOC level. In blood and lymphatic system disorders, HREL treatment demonstrated a significant ROR of 2.72 (95% CI 2.20, 3.37), higher than that of HREM treatment (ROR 2.35, 95% CI 1.77, 3.12). For skin and subcutaneous tissue disorders, both HREL and HREM treatments had RORs exceeding 2, at 2.07 (95% CI 1.79, 2.39) and 2.07 (95% CI 1.73, 2.47), respectively, indicating similar risk profiles for skin-related AEs with both treatments. In immune system disorders, HREL had an ROR of 2.05 (95% CI 1.53, 2.76), higher than that of HREM treatment (ROR 1.77, 95% CI 1.20, 2.61), suggesting a greater risk of immune system disorders with HREL. For ear and labyrinth disorders, HREM had an ROR of 2.62 (95% CI 1.58, 4.37), higher than that of HREL treatment (ROR 1.49, 95% CI 0.87, 2.58), indicating a stronger association of HREM with ear and labyrinth disorders. All signal details are shown in Table 4.

**TABLE 4:** Details of the SOC signals.

SOC	HREL case reports	ROR (95% CI)	PRR (95% CI)	Chisq	IC(IC025)	EBGM(EBGM05)	HREM case reports	ROR (95% CI)	PRR (95% CI)	Chisq	IC(IC025)	EBGM(EBGM05)
Hepatobiliary disorders	161	9.74 (8.29, 11.45)	9.01 (7.7, 10.54)	1,157.51	3.17 (2.94)	9.01 (7.87)	101	9.37 (7.65, 11.49)	8.71 (7.16, 10.6)	695.75	3.12 (2.83)	8.71 (7.35)
Blood and lymphatic system disorders	88	2.72 (2.2, 3.37)	2.64 (2.17, 3.21)	91.45	1.4 (1.1)	2.64 (2.21)	50	2.35 (1.77, 3.12)	2.3 (1.75, 3.03)	37.23	1.2 (0.8)	2.3 (1.81)
Skin and subcutaneous tissue disorders	206	2.07 (1.79, 2.39)	1.95 (1.7, 2.24)	101.46	0.97 (0.76)	1.95 (1.73)	137	2.07 (1.73, 2.47)	1.96 (1.68, 2.29)	67.73	0.97 (0.71)	1.96 (1.69)
Immune system disorders	45	2.05 (1.53, 2.76)	2.03 (1.51, 2.72)	23.78	1.02 (0.6)	2.03 (1.58)	26	1.77 (1.2, 2.61)	1.75 (1.21, 2.54)	8.49	0.81 (0.26)	1.75 (1.27)
Infections and infestations	182	1.8 (1.55, 2.1)	1.73 (1.51, 1.98)	59.2	0.79 (0.57)	1.73 (1.52)	107	1.58 (1.3, 1.93)	1.53 (1.28, 1.83)	21.07	0.62 (0.33)	1.53 (1.3)
Ear and labyrinth disorders	13	1.49 (0.87, 2.58)	1.49 (0.86, 2.58)	2.11	0.58 (-0.18)	1.49 (0.94)	15	2.62 (1.58, 4.37)	2.61 (1.57, 4.34)	14.9	1.38 (0.67)	2.6 (1.7)
Pregnancy, puerperium and perinatal conditions	11	1.37 (0.76, 2.48)	1.37 (0.76, 2.47)	1.1	0.45 (-0.37)	1.37 (0.83)	6	1.15 (0.52, 2.56)	1.15 (0.51, 2.57)	0.11	0.2 (-0.87)	1.15 (0.59)
Renal and urinary disorders	48	1.3 (0.98, 1.73)	1.29 (0.98, 1.7)	3.28	0.37 (-0.04)	1.29 (1.02)	22	0.89 (0.58, 1.35)	0.89 (0.59, 1.34)	0.3	−0.17 (-0.76)	0.89 (0.63)
Investigations	154	1.29 (1.09, 1.52)	1.26 (1.08, 1.47)	9.06	0.34 (0.1)	1.26 (1.1)	95	1.22 (0.99, 1.5)	1.2 (0.99, 1.46)	3.39	0.26 (-0.04)	1.2 (1.01)
Respiratory, thoracic and mediastinal disorders	103	1.09 (0.89, 1.33)	1.09 (0.9, 1.33)	0.73	0.12 (-0.17)	1.09 (0.92)	36	0.56 (0.4, 0.78)	0.58 (0.42, 0.79)	11.85	−0.8 (-1.27)	0.58 (0.44)
Eye disorders	41	1.02 (0.75, 1.39)	1.02 (0.76, 1.37)	0.01	0.03 (-0.41)	1.02 (0.79)	38	1.44 (1.04, 1.98)	1.42 (1.04, 1.94)	4.9	0.51 (0.05)	1.42 (1.09)
General disorders and administration site conditions	350	0.99 (0.88, 1.11)	0.99 (0.9, 1.09)	0.02	−0.01 (-0.17)	0.99 (0.9)	251	1.09 (0.95, 1.25)	1.07 (0.95, 1.2)	1.55	0.1 (-0.09)	1.07 (0.96)
Nervous system disorders	148	0.87 (0.74, 1.03)	0.88 (0.75, 1.03)	2.59	−0.18 (-0.42)	0.88 (0.77)	137	1.28 (1.07, 1.52)	1.25 (1.07, 1.46)	7.34	0.32 (0.07)	1.25 (1.08)
Metabolism and nutrition disorders	36	0.85 (0.61, 1.18)	0.85 (0.61, 1.19)	0.98	−0.24 (-0.71)	0.85 (0.64)	21	0.75 (0.49, 1.15)	0.75 (0.49, 1.15)	1.73	−0.41 (-1.02)	0.75 (0.53)
Endocrine disorders	4	0.8 (0.3, 2.12)	0.8 (0.3, 2.13)	0.21	−0.33 (-1.6)	0.8 (0.35)	4	1.2 (0.45, 3.21)	1.2 (0.45, 3.2)	0.13	0.26 (-1)	1.2 (0.53)
Gastrointestinal disorders	131	0.75 (0.63, 0.89)	0.77 (0.66, 0.9)	10.36	−0.39 (-0.64)	0.77 (0.66)	55	0.47 (0.36, 0.61)	0.49 (0.38, 0.63)	32.17	−1.03 (-1.42)	0.49 (0.39)
Vascular disorders	30	0.69 (0.48, 0.99)	0.7 (0.49, 1)	4	−0.52 (-1.03)	0.7 (0.52)	17	0.61 (0.38, 0.98)	0.61 (0.38, 0.98)	4.32	−0.71 (-1.38)	0.61 (0.41)
Cardiac disorders	31	0.59 (0.41, 0.84)	0.59 (0.41, 0.84)	8.89	−0.75 (-1.26)	0.59 (0.44)	15	0.44 (0.27, 0.74)	0.45 (0.27, 0.75)	10.31	−1.15 (-1.86)	0.45 (0.29)
Musculoskeletal and connective tissue disorders	46	0.41 (0.31, 0.55)	0.43 (0.32, 0.58)	37.51	−1.23 (-1.65)	0.43 (0.33)	47	0.65 (0.48, 0.87)	0.66 (0.5, 0.87)	8.66	−0.6 (-1.01)	0.66 (0.52)
Injury, poisoning and procedural complications	68	0.34 (0.27, 0.43)	0.36 (0.28, 0.46)	85.04	−1.47 (-1.82)	0.36 (0.3)	68	0.51 (0.4, 0.65)	0.54 (0.43, 0.68)	30.13	−0.9 (-1.25)	0.54 (0.44)
Psychiatric disorders	33	0.28 (0.2, 0.4)	0.3 (0.21, 0.42)	58.33	−1.75 (-2.24)	0.3 (0.22)	24	0.32 (0.21, 0.48)	0.33 (0.22, 0.49)	34.54	−1.6 (-2.17)	0.33 (0.24)
Neoplasms benign, malignant and unspecified (incl cysts and polyps)	6	0.11 (0.05, 0.24)	0.11 (0.05, 0.25)	44.53	−3.19 (-4.26)	0.11 (0.06)	NA	NA	NA	NA	NA	NA

### 3.3 Analysis by PT level

**FIGURE 3:**
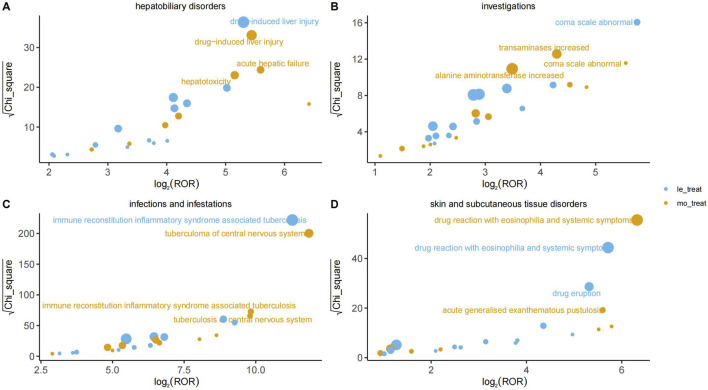
Comparison of Adverse Event Signals by Organ System: HREL vs. HREM.

At the PT level, Figure 3 visualizes the disproportionality analysis signals for key AEs across different organ systems (Panels A–D). In this analysis, the association strength is plotted on the X-axis as log_2_ (ROR), where values greater than 0 indicate a positive association. The statistical significance, measured by **√χ** ^**2**^, is plotted on the Y-axis, where higher values indicate a lower probability that the association occurred by chance. The bubble size is proportional to the report count for each event. A signal was considered significant and the corresponding AE was labeled on the plot only if it met the pre-defined thresholds for all four disproportionality analysis algorithms simultaneously (Table 2). All signal details are presented in Supplement Materials 1.

#### 3.3.1 Infections and infestations

The most prominent signal in this system organ class was for immune reconstitution inflammatory syndrome-associated tuberculosis (IRIS-TB), which demonstrated an exceptionally high ROR of 2,484.44 (95% CI 1600.57–3,856.40) in the HREL group. Significant signals were also observed for pathogen resistance (HREM: ROR = 40.54 (95% CI 20.22–81.28)) and cryptococcosis (HREL: ROR = 79.97 (95% CI 29.94–213.63)). These findings indicate a critical need for vigilant monitoring for opportunistic infections and the development of drug resistance during therapy with the target drug.

#### 3.3.2 Hepatobiliary disorders

Hepatobiliary disorders represented a major target for adverse drug reactions. A strong signal was consistently identified for drug-induced liver injury in both the HREL and HREM group (ROR = 39.48 (95% CI 28.38–54.91)) and ROR = 43.47 (95% CI 29.68–63.66), respectively). The signal for acute hepatic failure was particularly notable, with an ROR of 48.27 (95% CI 27.94–83.39) in the HREM group. Furthermore, significant signals for hepatitis (HREL: ROR = 17.52 (95% CI 10.36–29.65)) and hepatotoxicity (HREM: ROR = 35.61 (95% CI 21.74–58.32)) confirmed a substantial risk of hepatocellular injury.

#### 3.3.3 Nervous system disorders

Nervous system disorders included several highly specific signals with exceptionally elevated risks. The association for thalamic infarction was among the strongest observed across all PTs (HREL: ROR = 288.88 (95% CI 129.04–646.71); HREM: ROR = 520.22 (95% CI 246.20–1,099.23)). Similarly, extremely high risks were evident for central nervous system necrosis (HREL: ROR = 470.00 (95% CI 174.65–1,264.76)) and brain stem infarction (HREM: ROR = 184.88 (95% CI 69.11–494.60)), suggesting the potential for severe vascular or ischemic neurological events.

#### 3.3.4 Skin and subcutaneous tissue disorders

Among skin and subcutaneous tissue disorders, the most significant signal was for drug reaction with eosinophilia and systemic symptoms (DRESS), with an ROR of 79.88 (95% CI 58.50–109.07) in the HREM group. Other severe cutaneous adverse reactions, such as toxic epidermal necrolysis (HREL: ROR = 20.45 (95% CI 10.62–39.37)) and acute generalised exanthematous pustulosis (HREM: ROR = 48.47 (95% CI 24.17–97.17)), also demonstrated strong associations, underscoring the necessity for vigilance regarding serious skin reactions.

#### 3.3.5 Other system organ classes

In other system organ classes, the most outstanding signal was for paradoxical drug reaction, which exhibited remarkably high ROR values (HREL: ROR = 311.98 (95% CI 219.53–443.35); HREM: ROR = 564.12 (95% CI 405.81–784.19)). Drug resistance (HREM: ROR = 70.15 (95% CI 50.78–96.90)) and immune reconstitution inflammatory syndrome (HREL: ROR = 156.53 (95% CI 106.18–230.76)) were also key signals. Additionally, in the blood and lymphatic system, eosinophil count increased (HREL: ROR = 18.76 (95% CI 7.80–45.13)) suggested a potential for hypersensitivity-related reactions.

### 3.4 Subgroup analysis by age and gender

All signal details are shown in Supplement Materials 2 and Supplement Materials 3.

#### 3.4.1 Age stratification (≥60 vs. <60 years)

The elevated risk of IRIS-TB associated with HREL was dramatically higher in elderly patients (≥60 years, ROR 25810.17) compared to younger patients (<60 years, ROR 682.11). Notably, no significant IRIS-TB signal was detected for HREM in the elderly subgroup. Conversely, the risk of DILI and DRESS with HREM was more pronounced in elderly patients.

#### 3.4.2 Gender stratification

**FIGURE 4:**
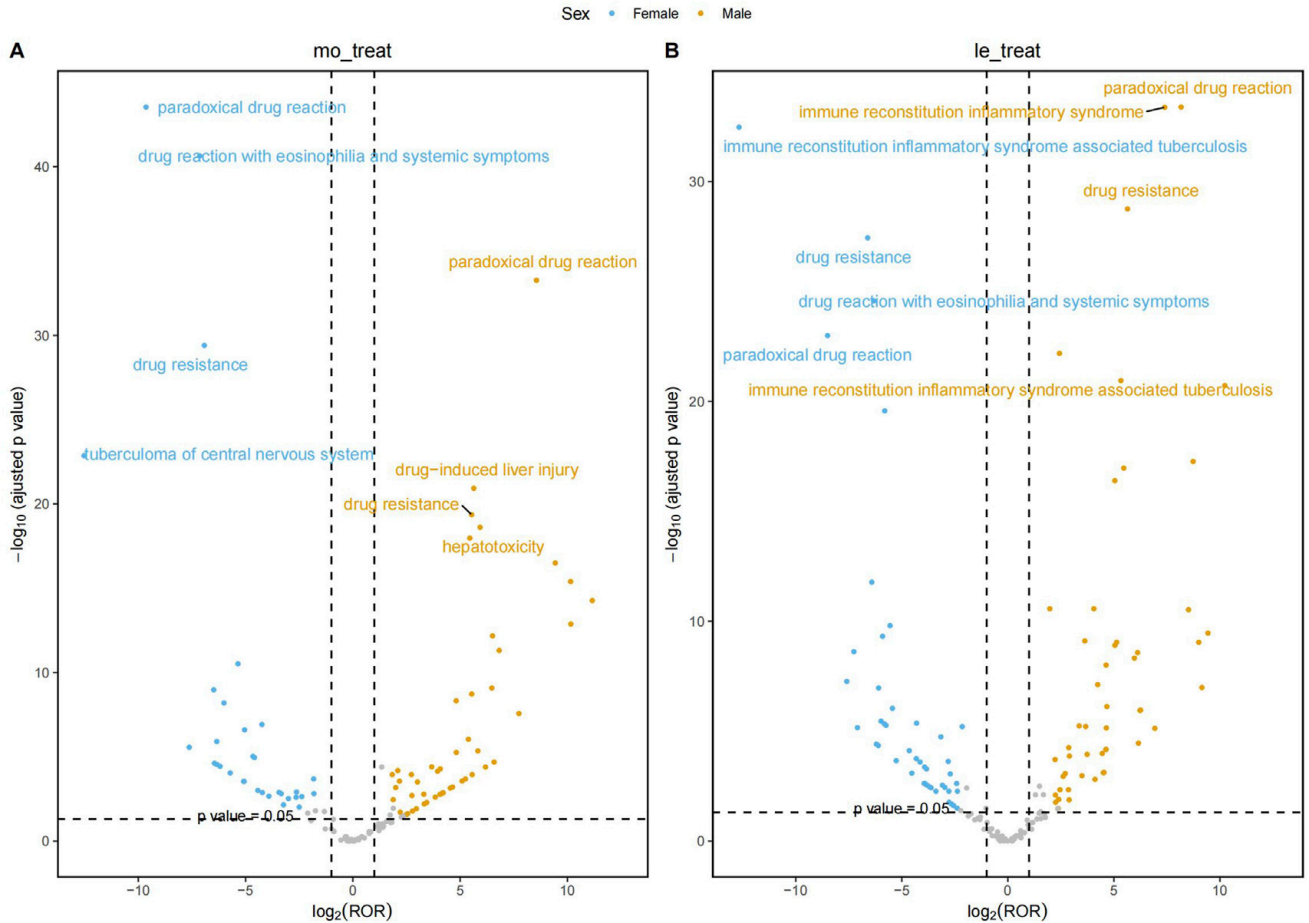
Adverse Event Risks Stratified by Gender: HREM and HREL.

At gender stratification, Figure 4 visualizes the results of the disproportionality analysis for key AEs in the HREM and HREL subgroups. The X-axis represents the log_2_-transformed ROR (log_2_ (ROR)). A vertical dashed line marks the threshold of log_2_ (ROR) = 1, which corresponds to the critical ROR value of 2 (since 2^1^ = 2) as defined in Table 2. The Y-axis represents the negative logarithm (base 10) of the p-value (-log_**10**_ (p-value)). A horizontal dashed line marks the conventional threshold for statistical significance, corresponding to a p-value of 0.05 (-log_10_ (0.05)≈1.3). The grey shaded area denotes the region where signals did not meet the dual criteria of both a statistically significant association (p-value <0.05) and a sufficiently strong association (ROR≥2). The gray points located above the dashed line represent signals that, while statistically significant (p < 0.05), did not meet the predefined thresholds of all four algorithms and thus were not considered robust signals.

The most prominent signal observed in female patients receiving HREL therapy was for IRIS-TB (ROR = 6,532.74, 95% CI: 3,338.12–12784.65), representing a 5.4-fold higher risk compared to male patients (ROR = 1,201.87). Concurrently, female patients demonstrated significantly elevated risk of drug-induced liver injury (ROR = 55.90) compared to males (ROR = 32.98). Among male patients treated with HREM, the risk of thalamic infarction was particularly notable (ROR = 691.55, 95% CI: 324.80–1,472.40), followed by brain stem infarction (ROR = 213.75). Regarding the HREM regimen, female patients demonstrated substantially higher risk of drug reaction with eosinophilia and systemic symptoms (ROR = 140.81) compared to male patients (ROR = 43.78). Concurrently, significantly stronger risk signals were observed in females for acute generalized exanthematous pustulosis (ROR = 89.53), jaundice (ROR = 33.06), and cholestasis (ROR = 21.52).

### 3.5 Time-to-onset analysis

**FIGURE 5:**
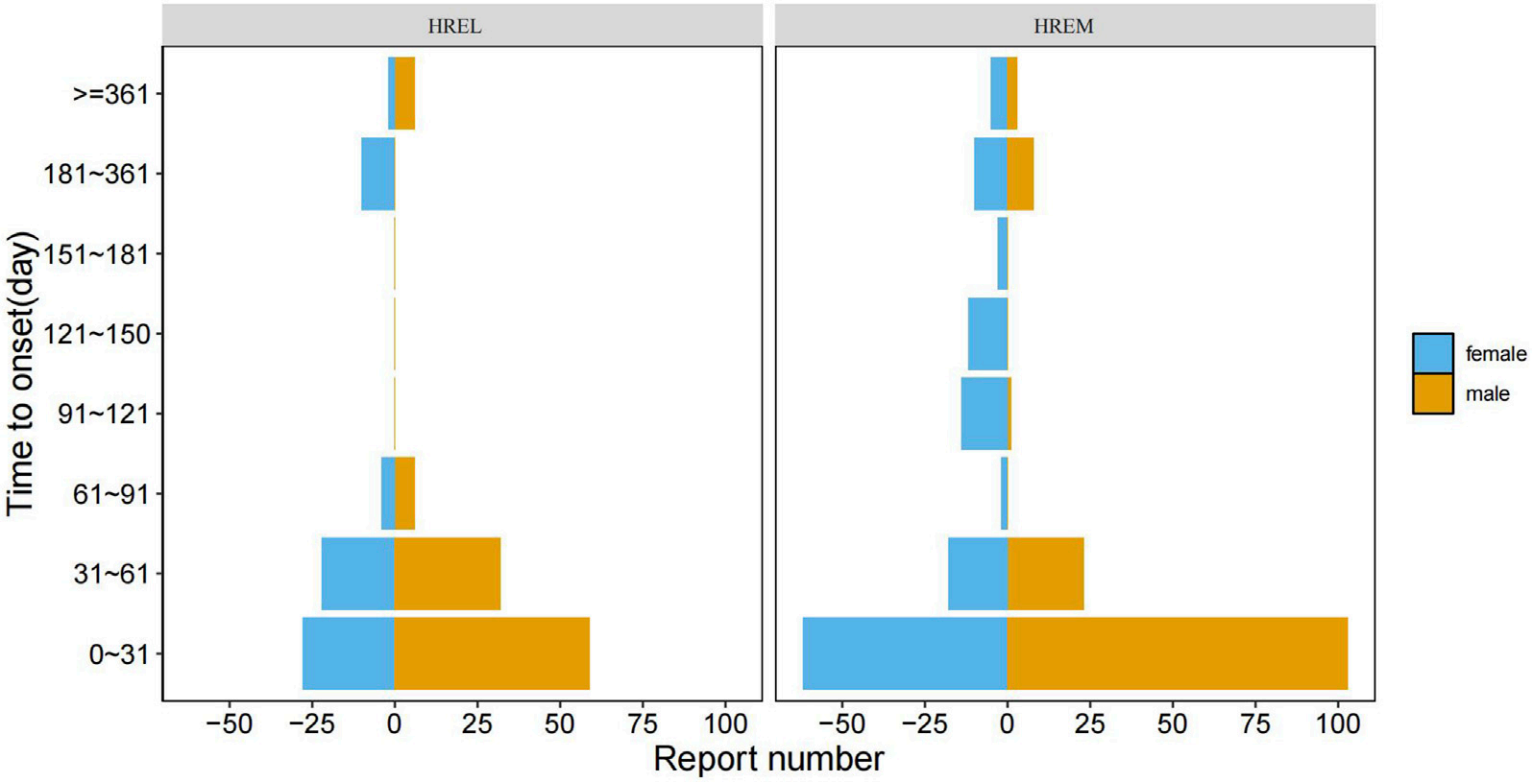
Gender Distribution in Time-to-Onset of Adverse Events: HREL vs. HREM.

**FIGURE 6:**
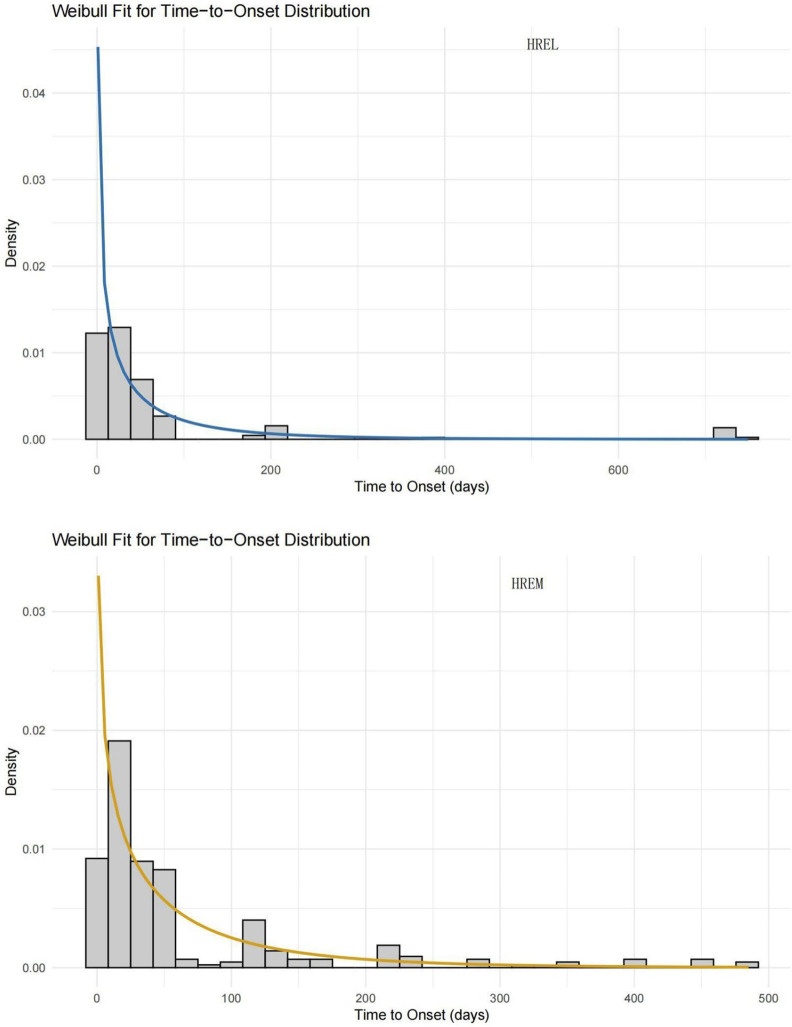
Weibull Fit for Time-to-Onset Distribution of Adverse Events: HREL and HREM.

The median time to onset (TTO) was shorter in the HREL group (18 days) compared to the HREM group (22 ays), with interquartile ranges of 4.75–45.50 and 10.00–54.00 days, respectively (Figure 5). Furthermore, we supplemented the Weibull distribution tests for the TTO analysis (Figure 6). The parameter estimates revealed a shape parameter (**β**) of 0.68 (95% CI: 0.609, 0.751) and a scale parameter (**α**) of 48.318 (95% CI: 37.107, 59.529) for the HREL group. For the HREM group, the values were **β** = 0.776 (95% CI: 0.706, 0.846) and **α** = 55.182 (95% CI: 45.906, 64.458). As the shape parameter **β** was less than 1 in both groups, AEs demonstrated a tendency to occur early in the treatment course.

## 4 Discussion

The substitution of pyrazinamide with fluoroquinolones in the treatment of drug-susceptible tuberculosis has long been widely practiced. However, aside from the well-recognized risk of QT interval prolongation, clinicians remain relatively uninformed about other adverse effects associated with this regimen. To our knowledge, this is the first pharmacovigilance study comparing AEs between HREL and HREM regimens. These insights contribute to the ongoing debate regarding the optimal choice of fluoroquinolone in tuberculosis treatment, especially for vulnerable populations such as the elderly.

### 4.1 Infections and infestations

IRIS-TB is a complex and severe complication primarily characterized by abnormal inflammatory responses following immune activation. It is an early complication observed in HIV-infected/AIDS patients with TB after initiating antiretroviral therapy (ART), marked by localized or systemic excessive inflammatory reactions (Ravimohan et al., 2015). In our study, we found a significantly increased risk of IRIS-TB associated with HREL treatment, particularly among younger patients and females, which warrants serious attention. This suggests a potential interaction between HREL and the dynamics of immune reconstitution. This phenomenon may be attributed to the immunomodulatory effects of the drug. Levofloxacin can reduce the release of pro-inflammatory cytokines such as IL-6, IL-8, and TNF-α via the TLR4/NF-κB pathway (Zusso et al., 2019). Over-suppression of pro-inflammatory signals may weaken macrophage activation and T-cell responses, leading to delayed antigen clearance. The residual antigens can continuously stimulate the immune system, triggering compensatory hyperactivation and driving IRIS-TB (Walker et al., 2015), especially in patients with a stronger immune system, such as younger individuals.

The higher proportion of IRIS-TB in females may be related to differences in hormone levels. For example, estrogen in female patients may enhance Th1-type immune responses (Dodd and Menon, 2022). Additionally, estrogen can induce the activation of M1-type macrophages via ERα, enhancing the inflammatory response to mycobacteria (Kim et al., 2018). The immunomodulatory effects of levofloxacin may synergize with these actions, leading to an uncontrollable inflammatory cascade. In contrast, moxifloxacin exhibited a lower IRIS-TB signal, which may be related to its pharmacokinetic properties (such as stronger tissue penetration) (Miravitlles, 2005) and higher receptor binding affinity (bacterial DNA topoisomerase) (Singh et al., 2022). These factors can rapidly reduce the bacterial load while avoiding excessive activation of the host immune response, thereby maintaining immune tolerance. For younger patients, females, and individuals with an active immune status, it is necessary to weigh the antimicrobial benefits of levofloxacin against the risk of IRIS-TB. It is recommended to prioritize drugs with lesser immune impact (such as moxifloxacin) and closely monitor inflammatory markers (such as CRP and IL-6).

### 4.2 Skin and subcutaneous tissue disorders

DRESS syndrome is a severe hypersensitivity reaction involving the skin and multiple organs. It is typically characterized by a rash and fever (Cardoso et al., 2011), accompanied by eosinophilia, atypical lymphocytosis, and multi-organ failure (Bocquet et al., 1996; Husain et al., 2013a; Husain et al., 2013b). Recent case series have shown that 15%–37% of DRESS syndrome cases may be caused by antimicrobial agents (Blumenthal et al., 2012). Among fluoroquinolones, moxifloxacin has been primarily associated with allergic reactions in previous studies (Sachs et al., 2006). Its metabolites may form covalent bonds with proteins, leading to immune system interactions (Andreu et al., 2009). The mechanism underlying skin allergies caused by fluoroquinolones is mainly IgE- and T-cell-dependent, with most reactions being IgE-mediated, such as urticaria and anaphylaxis (Zhang et al., 2022). However, DRESS syndrome induced by fluoroquinolone-containing regimens in tuberculosis treatment has rarely been studied.

Our study revealed that both HREL and HREM regimens are significantly associated with severe cutaneous adverse reactions, such as DRESS, but with distinct risk profiles. At the PT level, the ROR for DRESS associated with HREM was 79.88 (95% CI 58.50–109.07), significantly higher than that for HREL (ROR 52.41, 95% CI 38.31–71.71), consistent with prior findings. Subgroup analyses further indicated that elderly patients (>60 years) and females had a higher risk of skin-related AEs with HREM. In contrast, the risk signal for skin-related AEs with HREL was relatively lower, especially among elderly and male patients. Based on these results, a stratified management strategy is recommended in clinical practice. For patients with autoimmune diseases or those using immunomodulatory agents (e.g., corticosteroids), HREL should be prioritized to reduce the risk of DRESS. For patients who must receive HREM, enhanced skin monitoring is advised during the initial treatment period (especially the first 4 weeks), with a focus on erythema, vesicles, and systemic symptoms (e.g., fever, lymphadenopathy). In particular, for female and male patients, dynamic assessment of inflammatory markers (e.g., CRP, eosinophil count) may be necessary to evaluate risk.

### 4.3 Hepatobiliary disorders

Drug-induced liver injury (DILI) has emerged as a key concern for both treatment regimens, with similar reporting odds ratios (RORs) but distinct clinical implications. DILI associated with levofloxacin-containing regimens is more prevalent in females, potentially related to the regulation of estrogen-mediated oxidative stress pathways. Estrogen enhances mitochondrial reactive oxygen species (ROS) production by upregulating NADPH oxidase (Hsu et al., 2021) and inhibiting glutathione synthesis (Klinge, 2008), thereby increasing oxidative stress. Concurrently, levofloxacin increases ROS generation through multiple mechanisms, leading to mitochondrial membrane depolarization (Wei et al., 2022). Mitochondrial damage, a well-documented cause of widespread liver injury, has also been reported in hepatitis and cirrhosis (Nishikawa et al., 2014; Wang and Weinman, 2013). The ROS generated by these two mechanisms may act synergistically to cause hepatocyte mitochondrial damage and induce apoptosis. Although levofloxacin is primarily metabolized renally, liver function monitoring is still necessary for females receiving levofloxacin-containing regimens due to the potential risk. A retrospective cohort study based on a U.S. insurance claims database showed that both levofloxacin and moxifloxacin are associated with acute liver injury, but the risk is higher with levofloxacin compared to moxifloxacin (relative risk [RR] 3.2 vs. 2.3) (Kaye et al., 2014). Notably, moxifloxacin-containing regimens were found to have higher signals for severe outcomes such as acute liver failure. This difference may be related to the metabolic pathways of moxifloxacin. Moxifloxacin undergoes glucuronidation (mediated by UGT1A1/1A3) and biliary excretion, bypassing renal clearance (Annisa et al., 2022). These findings are consistent with clinical observations (Nori et al., 2004; Verma et al., 2009). These results underscore the importance of baseline liver function assessment and tailored monitoring strategies in drug-susceptible tuberculosis treatment, especially in high-risk populations with predisposing factors for liver injury.

### 4.4 Nervous system disorders

Our analysis identified significant neurological safety signals associated with both regimens, particularly concerning cerebrovascular events. The most notable associations were observed for thalamic infarction (HREM: ROR = 520.22; HREL: ROR = 288.88) and central nervous system necrosis (HREL: ROR = 470.00), with male patients showing heightened susceptibility to these severe complications. In 2013, the FDA required a black-box warning for these drugs to highlight the risk (US Food and Drug Administration FDA Drug Safety Communication, 2013). The underlying mechanisms may involve fluoroquinolone-induced magnesium chelation (Lindner et al., 2002) leading to hypomagnesemia and vasospasm, and GABA receptor inhibition potentially contributing to neuroexcitatory events (Grill and Maganti, 2011; Sanders et al., 2022). Additionally, the significant peripheral neuropathy signal with HREM (ROR = 7.76) aligns with previous case-control study. This study showed a significant increase in the incidence of peripheral neuropathy within 30 days of exposure to oral fluoroquinolones, with an additional 3% risk for each day of exposure, remaining significant up to 180 days post-exposure (Morales et al., 2019). These findings underscore the need for heightened clinical vigilance for neurological symptoms—particularly in male patients receiving HREM—including sensory disturbances, motor deficits, and acute cognitive changes. Prompt neurological assessment and consideration of regimen adjustment are recommended when such symptoms emerge during treatment.

### 4.5 Limitation

This analysis is constrained by the inherent biases of spontaneous reporting systems, including underreporting, incomplete clinical data, and confounding by indication. The overrepresentation of reports from specific regions (e.g., Japan and the U.S.) limits generalizability, and the lack of causality assessment precludes definitive conclusions. Furthermore, the route of administration was unspecified (“other”) in over 70% of reports for both regimens. Given that uneven regional distribution and differences in routes of administration may significantly influence drug safety profiles, this lack of detail warrants caution when interpreting and generalizing these conclusions. Finally, the absence of treatment duration and dosage details in FAERS hampers dose-response analyses.

## 5 Conclusion

For elderly patients (≥60 years), HREM may be a more suitable fluoroquinolone due to its lower risk of immune-mediated AEs such as IRIS-TB; however, clinicians should closely monitor for severe liver injury and cutaneous hypersensitivity reactions. HREL is more appropriate for younger patients (<60 years), but caution is needed regarding hepatotoxicity and IRIS-TB, especially in female patients. For males receiving HREM, increased vigilance for neurologic AEs is recommended. Future studies should prospectively validate these findings and explore mechanisms to optimize treatment strategies.

## Data Availability

The original contributions presented in the study are included in the article/supplementary material, further inquiries can be directed to the corresponding author.
